# The COVID-19 pandemic experience for patients with central nervous system tumors: Differences in patient-reported outcomes and practice recommendations

**DOI:** 10.1093/nop/npae067

**Published:** 2024-07-19

**Authors:** Amanda L King, Kayla N Roche, Elizabeth Vera, Valentina Pillai, Lily Polskin, Alvina A Acquaye-Mallory, Lisa Boris, Eric Burton, Anna Choi, Ewa Grajkowska, Heather E Leeper, Marissa Panzer, Marta Penas-Prado, Jennifer Reyes, Solmaz Sahebjam, Brett J Theeler, Jing Wu, Mark R Gilbert, Terri S Armstrong

**Affiliations:** Neuro-Oncology Branch, National Cancer Institute, National Institutes of Health, Bethesda, Maryland, USA; Neuro-Oncology Branch, National Cancer Institute, National Institutes of Health, Bethesda, Maryland, USA; Neuro-Oncology Branch, National Cancer Institute, National Institutes of Health, Bethesda, Maryland, USA; Frederick National Laboratory for Cancer Research, Leidos Biomedical Research, Inc., Frederick, Maryland, USA; Frederick National Laboratory for Cancer Research, Leidos Biomedical Research, Inc., Frederick, Maryland, USA; Neuro-Oncology Branch, National Cancer Institute, National Institutes of Health, Bethesda, Maryland, USA; Frederick National Laboratory for Cancer Research, Leidos Biomedical Research, Inc., Frederick, Maryland, USA; Neuro-Oncology Branch, National Cancer Institute, National Institutes of Health, Bethesda, Maryland, USA; Frederick National Laboratory for Cancer Research, Leidos Biomedical Research, Inc., Frederick, Maryland, USA; Neuro-Oncology Branch, National Cancer Institute, National Institutes of Health, Bethesda, Maryland, USA; Neuro-Oncology Branch, National Cancer Institute, National Institutes of Health, Bethesda, Maryland, USA; Frederick National Laboratory for Cancer Research, Leidos Biomedical Research, Inc., Frederick, Maryland, USA; Neuro-Oncology Branch, National Cancer Institute, National Institutes of Health, Bethesda, Maryland, USA; Neuro-Oncology Branch, National Cancer Institute, National Institutes of Health, Bethesda, Maryland, USA; Neuro-Oncology Branch, National Cancer Institute, National Institutes of Health, Bethesda, Maryland, USA; Department of Neurology, Uniformed Services University of the Health Sciences, Bethesda, Maryland, USA; Neuro-Oncology Branch, National Cancer Institute, National Institutes of Health, Bethesda, Maryland, USA; Neuro-Oncology Branch, National Cancer Institute, National Institutes of Health, Bethesda, Maryland, USA; Neuro-Oncology Branch, National Cancer Institute, National Institutes of Health, Bethesda, Maryland, USA

**Keywords:** anxiety, CNS tumors, COVID, depression, health-related quality of life, symptoms

## Abstract

**Background:**

This study explored differences in patient-reported outcomes (PROs) for patients with central nervous system (CNS) tumors during COVID, compared to pre-pandemic assessments, in light of impacted access to in-person care.

**Methods:**

Patient-reported outcomes (PROMIS-Anxiety and Depression Short-Forms, EQ-5D-3L, MDASI-BT/Spine, NeuroQoL-Perceived Cognitive Functioning) were collected from 149 participants on the Neuro-Oncology Branch Natural History Study seen during the first year of COVID between March 2020 and February 2021, which were compared to assessments collected pre-COVID. Paired sample *t*-tests and proportion tests (*z*-tests) were used to compare PROs with effect sizes reported using Hedges *g* and Cohen’s *h*. Logistic regression models with backwards selection were used to identify risk factors for high levels of depression and anxiety pre- and during COVID.

**Results:**

Participants were primarily male (54%) and Caucasian (84%) with a median age of 46 (range 20–79) and 66% had high-grade tumors. More patients reported moderate-severe depressive symptoms during the COVID year, compared to pre-COVID assessments (13% vs 8%, Cohen’s *h* = 0.17, *P* = .021), with modest increases in symptom burden and cognitive dysfunction reported as well. Logistic regressions revealed that during COVID, concurrent moderate-severe distress and low tumor grade predicted depression and anxiety, with psychotropic medication use also predicting depression while active treatment predicted anxiety.

**Conclusion:**

During COVID, patients experienced higher levels of depression, which has the potential to negatively influence treatment success and survival. Future work is needed to incorporate innovative tools and interventions that can be utilized remotely to identify and target mood disturbance in these vulnerable patients.

**Importance of the Study**

This is one of the few studies exploring how symptom burden and interference, mood disturbance, cognitive function, and health-related quality of life differed in a sample of central nervous system (CNS) tumor patients during the COVID-19 pandemic compared to pre-pandemic assessments. In addition, this sample is inclusive of several rare CNS tumor types, including spine tumors, which are typically not well represented within the neuro-oncology literature. The finding that CNS tumor patients remained highly symptomatic with a modest increase in depressive symptoms during the COVID-19 pandemic underscores the critical importance of developing screening and targeted interventions that are effective, yet adaptable, to pandemic-era restrictions. We suggest several options for future consideration to address this issue, including the use of telehealth, remote symptom monitoring, and expanded virtual therapies for targeting depression and anxiety symptoms within this patient population

Patients with central nervous system (CNS) tumors tend to be highly symptomatic.^1^ Some of the most commonly reported symptoms among brain tumor patients are fatigue, drowsiness, cognitive deficits such as problems remembering and difficulty speaking, disturbed sleep, and psychological distress.^2^ Among spine tumor patients, common symptoms are numbness or tingling, fatigue, weakness, and pain.^3^ Psychological distress, in particular, is remarkably elevated in patients with CNS tumors, compared to both the general population and other solid tumor patients^4,5^ with few non-pharmacological interventions available. The high physical and psychological symptom burden that CNS tumor patients face can adversely impact their quality of life, which was reported in a recent review where most patients with both high- and low-grade tumors reported such impairment.^6^ Unfortunately, equivalent data on symptom burden, psychological distress, and quality of life is often unavailable for rare brain and spine tumors, which remain underrepresented in the literature.

The COVID-19 pandemic and associated mitigation procedures significantly altered daily life with numerous consequences for patients related to health care, social functioning, and economic stability.^7^ Several studies have explored the impact of the COVID-19 pandemic on various aspects of quality of life in solid tumor patients; for example, a recent survey of mixed solid tumor patients (*N* = 260) found that, compared to a normative pre-pandemic sample, patients reported significantly impaired global quality of life, diminished cognitive and social functioning, worse insomnia, and increased financial difficulties during the COVID-19 pandemic.^8^ Other studies have prospectively examined the impact of the COVID-19 pandemic on the psychological health of patients with CNS tumors specifically, as well as their caregivers. Voisin et al. reported significant stress and anxiety related to fear of contracting COVID, treatment delays, and changes in healthcare administration in an international sample of patients with brain tumors and their caregivers.^9^ A prospective, multicenter study in patients with advanced cancer (*N* = 401) found that the incidence of anxiety and depression during the pandemic exceeded 30% with female gender, younger age, and longer estimated survival time being significant predictors for emotional distress.^10^ Additionally, Kim et al.^11^ utilized an online survey to assess the impact of COVID-related treatment-related changes (ie, delays, cancellations) on fear of cancer recurrence, anxiety, and depression in breast tumor patients. The authors found that changes related to the treatment plan as a result of COVID-19 were significantly associated with higher levels of depression for patients, with high levels of anxiety and fear of cancer recurrence also reported.^11^ Based on these findings in other cancer populations, we hypothesized that significant shifts in social, economic, and healthcare security during the COVID-19 pandemic may have negatively impacted patients with CNS tumors as well, which has yet to be explored.

The purpose of this study was to explore how symptom burden and interference, mood disturbance, cognitive function, and health-related quality of life differed in a sample of CNS tumor patients during the COVID-19 pandemic when compared to pre-pandemic assessments. In addition, this study aims to report on these characteristics in a sample inclusive of several types of rare brain and spine tumors that are typically not reported in the neuro-oncology literature.

## Materials and Methods

### Study Population

This descriptive analysis utilized data from the Natural History Study (NHS) conducted in the Neuro-Oncology Branch (NOB) at the National Institutes of Health (NIH). The NHS is a longitudinal, prospective trial that collects biological, clinical, and patient-reported outcomes (PROs) data for patients with CNS tumors across their illness trajectory (NCT02851706). Patients are deemed eligible for the NHS if they are at least 18 years of age, have a diagnosis of a primary CNS tumor (including brain and spine tumors), and are able and willing to give written informed consent. For the purpose of this analysis, comparisons were made between a “COVID year” sample (*N* = 149), which is composed of patient assessments from March 2020 to February 2021, and a “pre-COVID” sample (*N* = 149), which is composed of the last assessment prior to February 2020 for each patient seen during the COVID year. If patients had multiple evaluations during the COVID year, only the first evaluation was included in this analysis. Demographic and clinical characteristics for patients are updated at each clinical evaluation and PROs data are collected prior to the patient meeting with the clinicians to discuss their disease status.

### Instruments (PROs)

#### MD Anderson Symptom Inventory-Brain Tumor/Spine Tumor

The MD Anderson Symptom Inventory-Brain Tumor (MDASI-BT) and MD Anderson Symptom Inventory-Spine Tumor (MDASI-SP) instruments were developed and validated to assess symptom burden and interference in primary CNS tumor populations.^12,13^ The MDASI-BT assesses 6 symptom factor domains: affective, cognitive, neurologic, treatment-related, general disease, and gastrointestinal; the MDASI-SP assesses 4 symptom factor domains: disease, autonomic function, constitutional/treatment, and emotional. Both versions have 2 interference subscales: activity-related and mood-related. Each symptom and interference item are scored on a 0 to 10 scale, with higher numbers indicating higher severity. For both instruments, symptom items rated ≥5 and interference items rated ≥2 are considered moderate-severe.

#### PROMIS Anxiety and Depression Short-Forms

The Patient Reported Outcome Measurement Information System (PROMIS) Anxiety and Depression Short-Forms (version 8a) have been validated for the assessment of anxiety and depressive symptoms in diverse clinical populations.^14^ The PROMIS-Depression instrument assesses mood, views of self, and social cognition, while the PROMIS Anxiety instrument assesses self-reported fear, anxious misery, and hyperarousal. Responses to PROMIS items are scored and converted into a standardized *t*-scores with a score of 50 being the average for the general U.S. population*. T*-scores greater than 60 indicate a moderate-severe level of anxiety or depression.

#### NeuroQoL-Perceived Cognitive Function

The Quality of Life in Neurological Disorders (NeuroQoL) Cognitive Function Short-Form (8-item) is designed to measure perceived difficulties in cognitive abilities, including memory, attention, and decision-making, or the application of such abilities to everyday tasks^15^ and has been validated for use in individuals with neurologic disorders.^16^ Responses to NeuroQoL items are scored and converted into a standardized *t*-scores with a *t*-score of 50 being the average for the general US population. *T*-scores less than 40 indicate a moderate-severe level of cognitive dysfunction.

#### EuroQol-5 Dimension-3 Levels

The EuroQol-5 Dimension-3 Levels (EQ-5D-3L) is a measure of general health status that has been validated in a variety of clinical populations (including oncology)^17^ and is composed of 5 dimensions: mobility, self-care, usual activities, pain/discomfort, and anxiety/depression that each have 3 response options (no problems, moderate problems, and extreme problems/unable to).^18^ An EQ-5D-3L index score was calculated using U.S. population-based weights and reflects the patient’s perception of their own health: an index score of 1 indicates that the patient perceives their health as perfect, an index score of 0 indicates that the patient perceives their health as bad as death, and a negative index score indicates that the patient perceives their health as worse than death.^19^

### Statistical Methods

All statistical analyses were conducted with IBM SPSS Statistics version 28.^20^ Descriptive statistics were used to report patient demographic and clinical characteristics, as well as the questionnaire scores. Comparison of patient clinical characteristics and PROs between COVID and pre-COVID assessments was done using paired sample *t*-tests and proportion tests (*z*-tests) with effect sizes reported using Hedges *g* and Cohen’s *h*. Additionally, a logistic regression with backwards selection was performed to identify risk factors for high levels of depression and anxiety in patients seen during the COVID year. Statistical significance was set at *P* < .05 for all analyses.

## Results

### Sample Characteristics

There were 149 patients who had a clinical evaluation pre-COVID and during the COVID year, as shown in Tables 1 and 2. This group of CNS tumor patients was composed of 54% male, 84% White, and 8% Hispanic or Latino with a median age of 46 years (range 20–79) and 66% had high-grade tumors. Most patients (83%) had primary brain tumors with the most common diagnoses being glioblastoma (22%), anaplastic astrocytoma (13%), anaplastic ependymoma (11%), anaplastic oligodendroglioma (9%), and ependymoma (9%); however, over 30 CNS tumor types are represented in this diverse sample. Nearly 75% of patients seen during the COVID year had received 1 or more treatments prior to that visit and 58% reported at least 1 past tumor recurrence. The vast majority of COVID year visits were for patients on imaging surveillance (73%) and 54% had a Karnofsky Performance Status (KPS) score of 90 or greater, which indicated high functioning.

**Table 1.** Patient Characteristics of COVID Year Sample (*N* = 149)

Age	
Mean (SD)	47 (13)
Median (range)	46 (20–79)
	*N* (%)
Sex	
Female	68 (46)
Male	81 (54)
Race	
White	125 (84)
Black or African American	7 (5)
Asian	8 (5)
Unknown	8 (5)
Other	1 (1)
Ethnicity	
Hispanic or Latino	12 (8)
Tumor type	
Glioblastoma	33 (22)
Anaplastic astrocytoma	19 (13)
Anaplastic oligodendroglioma	14 (9)
Anaplastic ependymoma	16 (11)
Ependymoma	14 (9)
Astrocytoma	8 (5)
Myxopapillary ependymoma	9 (5)
Oligodendroglioma	8 (5)
Diffuse midline glioma	2 (1)
Atypical meningioma	1 (1)
Gliosarcoma	3 (2)
Medulloblastoma	2 (1)
Pilocytic astrocytoma	3 (2)
Anaplastic meningioma	3 (2)
Anaplastic pleomorphic xanthoastrocytoma	2 (1)
Papillary tumor of pineal region	2 (1)
Pleomorphic xanthoastrocytoma	1 (1)
Dysembryoplastic neuroepithelial tumor	1 (1)
High-grade glioma	1 (1)
Anaplastic pilocytic astrocytoma	1 (1)
Central neurocytoma	1 (1)
Hemangiopericytoma	1 (1)
Anaplastic glioneuronal tumor	1 (1)
Glioneuronal tumor	1 (1)
High-grade neuroepithelial tumor	1 (1)
Low grade glial neoplasm	1 (1)
Oligoastrocytoma	1 (1)
Tumor grade	
Grade 1	12 (8)
Grade 2	36 (24)
Grade 3	57 (38)
Grade 4	42 (28)
None assigned	2 (1)
Tumor location	
Brain	123 (83)
Spine	13 (9)
Brain + spine	13 (9)

SD = standard deviation.

**Table 2.** Clinical Status and History at Time of Evaluation

	COVID year	Pre-COVID
*N*	149	149
	*N* (%)	*N* (%)
Visit type^*^		
Clinic	44 (30)	149 (100)
Telehealth	88 (59)	0 (0)
Phone	17 (11)	0 (0)
On active treatment		
Yes	40 (27)	38 (26)
No	109 (73)	111 (74)
Imaging result^*^		
No image	6 (4)	19 (13)
No progression	107 (72)	120 (81)
Progression	25 (17)	6 (4)
Unsure if progression	11 (7)	4 (3)
KPS score		
40	1 (1)	0 (0)
50	4 (3)	2 (1)
60	5 (3)	4 (3)
70	13 (9)	13 (9)
80	17 (11)	24 (16)
90	55 (37)	61 (41)
100	25 (17)	44 (30)
Not assessed	29 (20)	1 (1)
Surgery		
1	71 (48)	76 (51)
2	41 (28)	41 (28)
≥3	37 (25)	32 (22)
Radiation therapy		
0	19 (13)	31 (21)
1	94 (63)	86 (58)
≥2	36 (24)	32 (22)
Treatments		
0	39 (26)	51 (34)
1	60 (40)	58 (39)
2	24 (16)	19 (13)
≥3	25 (17)	21 (14)
Recurrence		
0	63 (42)	79 (53)
1	43 (29)	36 (24)
2	18 (12)	13 (9)
≥3	25 (17)	21 (14)

KPS = Karnofsky Performance Status.

^*^ *P* < .05.

The 2 variables that differed significantly for patients during the COVID year compared to pre-COVID assessments were the type of visit and the imaging results. Given social distancing constraints related to the first year of COVID, 59% of patients were seen via telehealth, 11% were evaluated via telephone, and 30% were seen in-person in the NOB clinic, compared to 100% in-person clinical evaluations pre-COVID (Cohen’s *h =* 0.73, *P* < .001). The proportion of patients who had imaging results suggestive of progression was also significantly higher during the COVID year compared to pre-COVID (17% vs 4%, Cohen’s *h* = 0.13, *P* < .001).

### Main Findings

#### Symptom Burden and Interference

Table 3 details comparisons of symptom burden and interference in CNS tumor patients during and pre-COVID. For patients with brain tumors, the mean overall symptom burden was similar (1.6 vs 1.5, respectively) but the mean activity-related interference scores were 0.43 points higher during the COVID year (SD = 2.30, 95% CI = 0.03, 0.82, Hedges *g* = 0.18, *P* = .034), which indicates more activity-related interference with a small effect size. Spine tumor patients had a mean overall symptom burden score that was 0.27 points higher (SD = 0.63, 95% CI = 0.00, 0.55, Hedges *g* = 0.42, *P* = .050) and a mean disease-related symptoms score that was 0.45 points higher (SD = 0.82, 95% CI = 0.09, 0.80, Hedges *g* = 0.53, *P* = .015) during COVID, with medium effect sizes for both. The top 3 most reported moderate-severe symptoms for patients with brain tumors during the COVID year were fatigue (27%), drowsiness (20%), and difficulty remembering (26%), while spine tumor patients reported fatigue (44%), radiating spine pain (39%), pain (35%), and numbness/tingling (25%) most frequently, as shown in Supplementary Table 1.

**Table 3.** Symptom Burden and Interference (MDASI-BT and MDASI-SP) Assessments During and Pre-COVID

	COVID year	Pre-COVID
MDASI-BT		
*N*	134	134
	Mean (SD)	Mean (SD)
Overall symptom burden	1.6 (1.8)	1.5 (1.5)
Affective	2.2 (2.1)	2.1 (2.1)
Cognitive	2.1 (2.4)	1.8 (1.9)
Neurologic	1.5 (1.9)	1.2 (1.8)
Treatment-related	1.6 (2.1)	1.6 (1.9)
General disease	1.3 (1.8)	1.1 (1.6)
GI	0.6 (1.7)	0.7 (1.7)
Overall interference	2.2 (2.5)	2.0 (2.2)
Activity-related^*^	2.5 (2.9)	2.1 (2.4)
Mood-related	1.9 (2.4)	1.8 (2.3)
Moderate-severe overall interference	40%	43%
Moderate-severe activity-related	43%	41%
Moderate-severe mood-related	38%	35%

MDASI-SP		
*N*	23	23
	Mean (SD)	Mean (SD)
Overall symptom burden^*^	2.1 (1.4)	1.8 (1.3)
Disease-related^*^	3.1 (1.7)	2.6 (1.7)
Autonomic function	1.9 (2.5)	1.8 (2.4)
Constitutional/treatment-related	1.9 (2.5)	1.0 (1.3)
Emotional	1.2 (1.6)	1.7 (1.7)
Overall interference	2.5 (2.1)	2.3 (2.1)
Activity-related	2.9 (2.4)	2.5 (2.5)
Mood-related	2.2 (2.5)	2.1 (2.3)
Moderate-severe overall interference	62%	48%
Moderate-severe activity-related	65%	57%
Moderate-severe mood-related	46%	44%

GI = gastrointestinal; MDASI-BT = MD Anderson Symptom Inventory-Brain Tumor; MDASI-SP = MD Anderson Symptom Inventory-Spine Tumor; NOB = Neuro-Oncology Branch; SD = standard deviation.

^*^ *P* < .05.

#### Mood Disturbance and Cognition.

**Figure 1.**
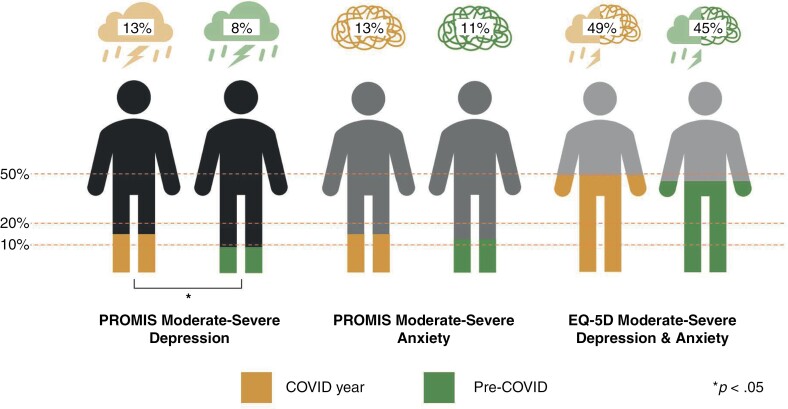
Frequency of moderate-severe PROMIS-Anxiety, PROMIS-Depression, and EQ-5D-3L Anxiety and Depression reports from CNS tumor patients during the COVID year and pre-COVID. Reference lines for 10% of sample, 20% of sample, and 50% of sample are provided. There was a significantly higher proportion of reports for moderate-severe depression reported on PROMIS during the COVID year (*P* = .021, Cohen’s *h* = 0.17) compared to the pre-COVID assessments, with no significant changes in PROMIS Anxiety or EQ-5D mood-related impact on quality of life.

During the COVID year, 13% of patients reported anxiety or depression at the moderate-severe level and 7% had co-occurrence of these conditions. Compared to pre-pandemic assessments, there was a significantly higher proportion of patients who reported moderate-severe depression during COVID (13% vs 8%, respectively, Cohen’s *h* = 0.17, *P* = .021), demonstrating a small effect size. Also, the mean *t*-score for the PROMIS-Depression instrument was 1.8 points higher during COVID, indicating a higher severity of depression, compared to pre-COVID measurements. There were no significant differences in the proportion of patients reporting moderate-severe anxiety during COVID compared to pre-COVID and mean *t*-scores were similar. Figure 1 illustrates differences in proportions of patients reporting mood disturbance during and pre-COVID.

With regards to cognitive function, there was a significantly higher proportion of patients who reported moderate-severe cognitive issues during COVID (24% vs 18%, respectively, Cohen’s *h* = 0.17, *P* = .050), which showed a small effect size. The mean *t*-score on the NeuroQoL instrument was 1.1 points lower during the COVID year, indicating worse cognitive function, compared to pre-pandemic assessments for patients. Additional details about changes in mood disturbance and cognitive function during COVID can be found in Supplementary Table 2.

#### Health-Related Quality of Life

There were no significant changes in the proportion of patients reporting issues with mobility, self-care, usual activities, pain/discomfort, or anxiety/depression on the EQ-5D-3L during COVID and the overall health index score was similar compared to pre-COVID assessment (0.83 vs 0.82, respectively). Additional details about quality of life reporting for patients with CNS tumors are shown in Supplementary Table 3.

### Regression Analyses

Logistic regressions with backwards selection were used to identify risk factors for moderate-severe levels of depression and anxiety in patients seen during the COVID year and pre-COVID, with final reduced models shown in Tables 4 and 5, with additional regression model information reported in Supplementary Tables 5–8. Independent variables in the models included age at the time of clinical evaluation, sex, race, ethnicity, current tumor grade, treatment status, KPS, progression status, prior recurrence, use of psychotropic medications, and co-occurrence of moderate-severe fatigue, distress, or disturbed sleep. Psychotropic medication use was reported by approximately 35% of patients, both during COVID and pre-COVID, which included medications such as antidepressants, anxiolytics, antipsychotics, mood stabilizers, and stimulants. The dependent variable in the regression models was the presence of moderate-severe depression or anxiety on the PROMIS instrument, defined by a *t*-score ≥ 60, as previously mentioned. The sample sizes for regression analyses performed during the COVID year were slightly smaller (*N* = 102) compared to the pre-COVID analyses (*N* = 128) due to missing data for predictor variables.

**Table 4.** Stepwise Logistic Regression Reduced Models Identifying Significant Predictors for Moderate-Severe Depression for Patients Seen During (*N* = 102) and Pre-COVID Year (*N* = 128)

During COVID				
**Variable**	**Level**	OR	95% CI	Sig
Current tumor grade	Low grade (1 or 2)	14.12	2.15, 92.59	**0.006**
	High grade (3 or 4)	1.00		
MS distress	Present	25.72	4.24, 155.87	**<0.001**
	Not present	1.00		
Psychotropic medication use	Yes	5.52	1.14, 26.79	**0.034**
	No	1.00		

Pre-COVID				
**Variable**	**Level**	OR	95% CI	Sig
MS distress	Present	30.46	5.18, 179.08	**<0.001**
	Not present	1.00		
Psychotropic medication use	Yes	9.87	1.59, 61.40	**0.014**
	No	1.00		

MS = moderate-severe; OR = odds ratio; Sig = significance level. Bolded Sig values indicate *P*-values <.05.

**Table 5.** Stepwise Logistic Regression Reduced Models Identifying Significant Predictors for Moderate-Severe Anxiety for Patients Seen During and Pre-COVID Year (*N* = 128)

During COVID				
**Variable**	**Level**	OR	95% CI	Sig
Current tumor grade	Low grade (1 or 2)	5.57	1.12, 27.61	**0.035**
	High grade (3 or 4)	1.00		
Active treatment at time of visit	Yes	5.82	1.05, 32.33	**0.044**
	No	1.00		
MS distress	Present	10.57	2.27, 49.17	**0.003**
	Not present	1.00		

Pre-COVID				
**Variable**	**Level**	OR	95% CI	Sig
MS distress	Present	9.79	3.12, 30.75	**<0.001**
	Not present	1.00		

MS = moderate-severe; OR = odds ratio; Sig = significance level. Bold sig values indicate variables found to be significant predictors in regression models. Bolded Sig values indicate *P*-values <.05.

#### Depression

A logistic regression among patients with all available variables yielded a model with current tumor grade, co-occurrence of moderate-severe distress, and psychotropic medication use influencing the likelihood of having moderate-severe depressive symptoms during the COVID year. During COVID, the odds of clinically significant depression increased 26-fold with co-occurrence of moderate-severe distress, 14-fold for those with low-grade tumors, and 6-fold for patients taking psychotropic medications. Pre-COVID, the predictors for depression were similar, apart from current tumor grade, with the odds of clinically significant depression increasing 31-fold with co-occurrence of moderate-severe distress and 10-fold for those taking psychotropic medications.

#### Anxiety

A logistic regression among patients with all available variables yielded a model with current tumor grade, active treatment at time of visit, and co-occurrence of moderate-severe distress influencing the likelihood of having moderate-severe anxiety symptoms during the COVID year. During COVID, the odds of clinically significant anxiety increased 11-fold with co-occurrence of moderate-severe distress and 6-fold for those with low-grade tumors and on active treatment. Pre-COVID, the only significant predictor for anxiety was co-occurrence of moderate-severe distress, which increased likelihood of clinically significant anxiety 10-fold.

## Discussion

Key findings from this study demonstrate that patients with CNS tumors who were seen during COVID remained highly symptomatic with a modest increase in depressive symptoms reported during this time. Increased mood disturbance during the first year of the pandemic may be attributed to novel stressors in the form of changing role responsibilities, social distancing, and limited healthcare access. In a recent study of breast cancer patients, 33% reported additional stress due to increased responsibilities because of the pandemic, 19% reported additional stress due to difficulty obtaining help or social support, and 19% reported additional stress due to postponement of cancer treatment.^21^ In this study, a higher level of concern related to COVID-19 was significantly associated with greater anxiety and depression, as well as insomnia and fear of cancer recurrence. Similarly, in a sample of Korean mixed-cancer patients, nearly half of the sample reported meaningful functional impairments related to work, home management, interpersonal relationships, and leisure activities during the pandemic.^22^ These functional impairments were predicted by disruptions in healthcare service utilization, high depression levels, anxiety regarding the viral epidemic, fear of COVID surpassing that related to their cancer, and low resilience. In addition, a recent review and meta-analysis of studies examining stress, anxiety, and depression during the COVID-19 pandemic in the general population found that individuals were slightly more affected by depression (33.7%), compared to stress and anxiety (29.6% and 31.9%, respectively), which aligns with our findings of higher depression levels.^23^ This may be the result of the COVID-19 pandemic specifically diminishing factors typically thought to be protective against depression, such as social support, sense of purpose, and socioeconomic stability, but further research is needed to clarify the specific aspects of the pandemic that can impact mood for cancer patients.

The regression analyses identified several predictors for moderate-severe levels of depression and anxiety during the COVID year and pre-pandemic. Prior to the pandemic, the co-occurrence of moderate-severe levels of distress predicted high levels of both depression and anxiety, with use of psychotropic medications also a predictor for depression. Distress is often conceptualized as existing along a continuum, ranging from normal adjustment to life stressors progressing to adjustment disorders and diagnosable anxiety and depressive disorders on the severe end of the spectrum.^24^ During COVID, the presence of a low-grade tumor predicted moderate-severe levels of depression and anxiety, which has been reported in past work in this population.^25^ While a lower tumor grade is associated with a more favorable prognosis, those patients may still experience significant symptom burden and functional deficits as a result of the tumor and/or treatment, which may adversely impact their psychological health,^6^ particularly if they lack buffering resources.^26,27^ The reason why this variable was only a significant predictor during the pandemic is unclear, but it is plausible that the ability for patients to effectively cope with their disease may have been hampered during this time. Use of psychotropic medications was a predictor for high levels of depression during COVID, which suggests that those with preexisting depression continued to have difficulties during that year. Additionally, being on active treatment predicted high levels of anxiety during COVID, which may reflect the more frequent neuroimaging during treatment and heightened “scanxiety” during an already stressful time.

Increased mood disturbance during the COVID-19 pandemic may have several important implications for the health and well-being of patients with CNS tumors. For example, distressed patients may be less inclined to self-manage their disease and symptoms. One study found that during the COVID-19 pandemic, breast cancer patients and survivors who experienced a deterioration in emotional functioning were less likely to contact their clinicians.^28^ Reluctance to contact clinicians may negatively impact the reporting of new or worsening symptoms and may also hinder the receipt of guidance on how to effectively manage such issues. Furthermore, it is well documented that cancer patients with depression are less likely to adhere to treatment plans and engage in pro-health behaviors, such as maintaining a healthy diet and exercise regimen and practicing good sleep hygiene.^29^ These effects may be amplified by pandemic-specific limitations that discourage individuals from leaving the home, further affecting overall health and well-being.

One key difference for patients during the COVID year, compared to pre-COVID assessments, is that a higher proportion of individuals had progression at their clinic visit, which may have contributed, at least in part, to the modest increases observed in symptom burden, mood disturbance, and cognitive dysfunction. Patients with spine tumors had worsening of disease-related symptoms, such as pain and radiating spine pain, while brain tumor patients had worsening of activity-related symptoms, which has been shown to predict recurrence in prior research.^30^ Given that patients in this sample completed their questionnaires prior to discussion with clinicians about their MRI results, they were not aware of the tumor progression at the time of completion, though patients may have suspected the results if experiencing significant changes in their symptoms. Interestingly, there is accumulating evidence across cancer populations that the presence of clinically significant mood disorders, particularly depression, can directly promote tumor progression and metastasis via excess adrenergic signaling and immune dysregulation, which can ultimately worsen overall survival.^31–33^ As such, the mentality is shifting away from viewing mood disorders as merely an expected side effect of having cancer and going through treatment, but instead considering anxiety and depression as important risk factors that can directly affect tumor behavior and response to treatments. Further work is needed in this area for patients with CNS tumors so that we can better identify at-risk patients prospectively from the time of diagnosis and provide targeted interventions that may improve their clinical outcomes.

Given that cancer patients more frequently interact with the healthcare system, substantial changes in healthcare delivery related to the pandemic, such as the transition to telehealth, may have disproportionately impacted this vulnerable population. One study focusing on the use of telemedicine in a neurosurgery clinic found that although satisfaction with telehealth was relatively high, difficulties did arise, including initial set-up for first-time telemedicine users, difficulty navigating the telehealth platform, management of patients without access to appropriate technology, difficulties with physical examination, and informality undermining the typical patient–clinician dynamic that is often critical to developing a therapeutic relationship.^34^ On one hand, telemedicine may improve healthcare access for some, especially for individuals who may struggle with overcoming the barrier of in-person clinic visits (such as severely depressed or fatigued individuals or those with social, economic, and geographical barriers to travel to specialized centers). Conversely, telehealth may restrict access for others, particularly for those with limited access to or knowledge of the technology, as well as for individuals who may benefit from building a meaningful in-person connection with their clinical team, which, again tends to benefit patients with significant mood disturbance.

Due to the high symptom burden that CNS tumor patients experience throughout their illness trajectory, it may be valuable to consider screening, interventions, and self-management strategies that can be completed remotely via telehealth platforms, particularly if future pandemics or health crises occur that require similar mitigation strategies. In recent work by Jones et al., the authors proposed a series of initiatives that may promote effective care for cancer patients and survivors in response to the COVID-19 pandemic.^35^ Adoption of remote symptom monitoring for patients is one recommendation that may improve targeted care, even in spite of reduced in-person visits.^35^ The NOB has recently led the development of a smartphone application, called My STORI, which empowers patients with CNS tumors to track and self-manage common symptoms that they experience throughout their illness trajectory. Such web-based symptom monitoring initiatives have shown promise in increasing cancer patient survival when compared to standard imaging surveillance alone^36^ and may help improve communication between patients and neuro-oncology clinicians, as well as promote patient self-efficacy in managing their symptoms.^37^

Jones et al. also highlighted the importance of addressing psychosocial needs in the wake of the pandemic by promoting effective screening and expansion of mental health services that can be accessed through phone and telemedicine platforms.^27^ One potentially promising psychosocial support program for patients with CNS tumors is the Managing Cancer and Living Meaningfully (CALM) program, which was developed as in-person initiative by Rodin et al. but is currently being piloted in the NOB as a remotely conducted intervention for patients with CNS tumors (NCT04852302).^38^ This program supports an individualized therapeutic relationship, with emphasis on symptom management, communication, relationships, spiritual well-being, and mortality concerns; previous studies have shown CALM to significantly reduce depression symptoms in advanced cancer patients.^29^ In addition, use of a remote virtual reality-based relaxation intervention that can target distress and anxiety symptoms at the time of clinical evaluations is being explored within the CNS tumor population.^39^ Both of these interventions are particularly relevant given pandemic-era challenges and represent important steps toward empowering patients to independently manage psychological symptoms in a remote setting.^40^ Ultimately, it may be prudent for clinicians to arrange more frequent follow-up with cancer patients who have preexisting mood disturbances in order to detect worsening of psychological health earlier so appropriate treatment can be sought.

### Limitations

There are limitations to the present study that may have impacted findings. In the early stages of the COVID-19 pandemic, the NOB transitioned to only evaluating patients who urgently needed treatment or were actively enrolled in a therapeutic clinical trial. As a result, the proportion of patients in the COVID year sample who received a disease progression result on imaging is likely higher than what is typical for patients seen prior to the pandemic. We also assessed changes in PROs over 2 timepoints (one pre-COVID assessment and one assessment during COVID), rather than longitudinally over several visits, which would have allowed more robust assessment of symptom trajectories. Unfortunately, only a small proportion of the sample was seen more than 1 time during the COVID year, which precluded that type of longitudinal analysis. Additionally, changes identified in PROs during COVID had mostly small effect sizes, therefore, the clinical impact on the patients may not have been significant. Lastly, the NIH is a specialty research institution that focuses entirely on clinical trials, therefore patients seen by the NOB may not be fully representative of patients with CNS tumors seen elsewhere.

## Conclusion

This study demonstrates that during the COVID-19 pandemic, patients with CNS tumors continued to have a high symptom burden and reported an increase in depressive symptoms, with a higher proportion of patients experiencing progression during this time. Although specific mechanisms predisposing individuals to an increased risk for mood disturbance have yet to be fully elucidated in this population, this work underscores the need for effective, pandemic-era interventions for screening, targeting, and improving depression and anxiety symptoms in order to mitigate influence on clinical outcomes. Pursuing future work along these directions will be foundational for improving psychological health and quality of life for this patient population.

## Supplementary material

Supplementary material is available online at *Neuro-Oncology Practice* (https://academic.oup.com/nop/).

npae067_suppl_Supplementary_Materials

## References

[1] Rogers J , E V, Acquaye A, et al Living with a central nervous system (CNS) tumor: findings on long-term survivorship from the NIH Natural History Study. Neurooncol Pract. 2021;8(4):460–474. 34277024 10.1093/nop/npab022 PMC8278352

[2] Armstrong TS , Vera-Bolanos E, Acquaye A, et al The symptom burden of primary brain tumors: evidence for a core set of tumor- and treatment-related symptoms. Neuro Oncol. 2016;18(2):252–260. 26289592 10.1093/neuonc/nov166 PMC4724180

[3] Acquaye A , Vera E, Gilbert M, Armstrong T. Clinical presentation and outcomes for adult ependymoma patients. Cancer. 2017;123(3):494–501. 27679985 10.1002/cncr.30355 PMC7886181

[4] Randazzo D , Peters K. Psychosocial distress and its effects on the health-related quality of life for primary brain tumor patients. CNS Oncol. 2018;5(4):241–249. 10.2217/cns-2016-0010 PMC6040083 27397796

[5] Zabora J , Brintzenhofe-Szoc K, Curbow B, Hooker C, Piantadosi S. The prevalence of psychological distress by cancer site. Psychooncology. 2001;10(1):19–28. 11180574 10.1002/1099-1611(200101/02)10:1<19::aid-pon501>3.0.co;2-6

[6] Taphoorn MJ , Sizoo EM, Bottomley A. Review on quality of life issues in patients with primary brain tumors. Oncologist. 2010;15(6):618–626. 20507891 10.1634/theoncologist.2009-0291 PMC3227985

[7] Haleem A , Javaid M, Vaishya R. Effects of COVID-19 pandemic in daily life. Curr Med Res Pract. 2020;10(2):78–79. 32292804 10.1016/j.cmrp.2020.03.011 PMC7147210

[8] Ciazynska M , Pabianek M, Szczepaniak K, et al Quality of life of cancer patients during coronavirus disease (COVID-19) pandemic. Psychooncology. 2020;29(9):1377–1379. 32779778 10.1002/pon.5434 PMC7323427

[9] Voisin M , Oliver K, Farrimond S, et al Brain tumors and COVID-19: the patient and caregiver experience. Neurooncol Adv. 2020;2(1):vdaa104. 32989433 10.1093/noajnl/vdaa104 PMC7499687

[10] Obispo-Portero B , Cruz-Castellanos P, Jimenez-Fonseca P, et al Anxiety and depression in patients with advanced cancer during the COVID-19 pandemic. Support Care Cancer. 2022;30(4):3363–3370. 34993652 10.1007/s00520-021-06789-3 PMC8735888

[11] Kim S , Kim S. Do COVID-19-related treatment changes influence fear of cancer recurrence, anxiety, and depression in breast cancer patients? Cancer Nurs. 2022;45(2):E628–E638. 33654008 10.1097/NCC.0000000000000937

[12] Armstrong TS , Mendoza T, Gring I, et al Validation of the M.D. Anderson Symptom Inventory Brain Tumor module (MDASI-BT). J Neurooncol. 2006;80(1):27–35. 16598415 10.1007/s11060-006-9135-z

[13] Armstrong T , Gning I, Mendoza T, et al Reliability and validity of the MD Anderson Symptom Inventory–Spine Tumor module. J Neurosurg Spine. 2010;12(4):421–430. 20367379 10.3171/2009.10.SPINE0943

[14] Schalet B , Pilkonis P, Yu L, et al Clinical validity of PROMIS® Depression, Anxiety, and Anger across diverse clinical samples. J Clin Epidemiol. 2016;73:119–127. 26931289 10.1016/j.jclinepi.2015.08.036 PMC4928679

[15] NINDS. User manual for the quality 2017. https://www.sralab.org/sites/default/files/2017-06/Neuro-QOL_User_Manual_v2_24Mar2015.pdf. Accessed March 26, 2024.

[16] Cella D , Lai J, Nowinski C, et al Neuro-QoL: brief measures of health-related quality of life for clinical research in neurology. Neurology. 2012;78(23):1860–1867. 22573626 10.1212/WNL.0b013e318258f744 PMC3369516

[17] Zeng X , Sui M, Liu B, et al Measurement properties of the EQ-5D-5L and EQ-5D-3L in six commonly diagnosed cancers. Patient. 2021;14(2):209–222. 33123985 10.1007/s40271-020-00466-z

[18] The EuroQoL Group. EuroQoL: a new facility for the measurement of health-related quality of life. Health Policy. 1990;16(3):199–208. 10109801 10.1016/0168-8510(90)90421-9

[19] Shaw J , Johnson J, Coons S. US valuation of the EQ-5D health states: development and testing of the D1 valuation model. Med Care. 2005;43(3):203–220. 15725977 10.1097/00005650-200503000-00003

[20] IBM Corp. IBM SPSS Statistics forWindows. In: Corp. I, ed. Armonk, NY: IBM; 2021.

[21] Massicotte V , Ivers H, Savard J. COVID-19 pandemic stressors and psychological symptoms in breast cancer patients. Curr Oncol. 2021;28(1):294–300. 33430131 10.3390/curroncol28010034 PMC7903274

[22] Kim K , Kim H, Lee J, et al Functional impairments in the mental health, depression, and anxiety related to the viral epidemic and disruption in the healthcare service utilization among cancer patients in the COVID-19 pandemic era. Cancer Res Treat. 2021;54(3):671–679. 34583461 10.4143/crt.2021.585 PMC9296928

[23] Salari N , Hosseinian-Far A, Jalali R, et al Prevalence of stress, anxiety, and depression among the general population during the COVID-19 pandemic: a systematic review and meta-analysis. Global Health. 2020;16(1):57. 32631403 10.1186/s12992-020-00589-w PMC7338126

[24] Schuyler D. Cognitive therapy for adjustment disorder in cancer patients. Psychiatry. 2004;1(1):20–23. 21197372 PMC3012613

[25] Arnold S , Forman L, Brigidi B, et al Evaluation and characterization of generalized anxiety and depression in patients with primary brain tumors. Neuro Oncol. 2008;10(2):171–181. 18314416 10.1215/15228517-2007-057 PMC2613819

[26] Johansson M , Ryden A, Finizia C. Mental adjustment to cancer and its relation to anxiety, depression, HRQL and survival in patients with laryngeal cancer: a longitudinal study. BMC Cancer. 2011;11(283). 10.1186/1471-2407-11-283 PMC3136424 21718478

[27] Myovic M , Block S. Psychiatric disorders in advanced cancer. Cancer. 2007;110(8):1665–1676. 17847017 10.1002/cncr.22980

[28] Bargon C , Batenburg M, van Stam L, et al Impact of the COVID-19 pandemic on patient-reported outcomes of breast cancer patients and survivors. JNCI Cancer Spectr. 2021;5(1):pkaa104. 33437925 10.1093/jncics/pkaa104 PMC7665619

[29] DiMatteo M , Haskard-Zolnierek K. Impact of depression on treatment adherence and survival from cancer. In: Kissane D, Maj M, Sartorius N, eds. Depression and Cancer. Oxford, UK: John Wiley & Sons, Ltd; 2011.

[30] Armstrong T , Vera-Bolanos E, Gning I, et al The impact of symptom interference using the MD Anderson Symptom Inventory-Brain Tumor module (MDASI-BT) on prediction of recurrence in primary brain tumor patients. Cancer. 2011;117(14):3222–3228. 21264841 10.1002/cncr.25892

[31] Otto-Meyer S , Lumibao J, Kim E, et al The interplay among psychological distress, the immune system, and brain tumor patient outcomes. Curr Opin Behav Sci. 2019;28:44–50. 31049368 10.1016/j.cobeha.2019.01.009 PMC6487487

[32] Lutgendorf S , Costanzo E, Sood A. Biobehavioral influences on tumor progression. In: Segerstrom S, ed. Psychoneuroimmunology and Cancer. Oxford, UK: Oxford Academic; 2012:341–368.

[33] Zhang R , Wang J, Zhang P, Zheng Z, Miao R. Pancreatic cancer progression and mortality predicted by depression and anxiety: a systematic review and meta-analysis protocol. Psychiatry. 2023;14. 10.3389/fpsyt.2023.1266502 PMC10808776 38274428

[34] Blue R , Yang A, Zhou C, et al Telemedicine in the era of coronavirus disease 2019 (COVID-19): a neurosurgical perspective. World Neurosurg. 2019;139:549–557. 10.1016/j.wneu.2020.05.066 PMC7229725 32426065

[35] Jones J , Saeed H, Katz M, et al Readdressing the needs of cancer survivors during COVID-19: a path forward. J Natl Cancer Inst. 2021;113(8):955–961. 33367655 10.1093/jnci/djaa200 PMC7799033

[36] Denis F , Basch E, Septans A, et al Two-year survival comparing web-based symptom monitoring versus routine surveillance following treatment for lung cancer. JAMA. 2019;321(3):306–307. 30667494 10.1001/jama.2018.18085 PMC6439676

[37] Basch E , Stover A, Schrag D, et al Clinical utility and user perceptions of a digital system for electronic patient-reported symptom monitoring during routine cancer care: findings from the PRO-TECT trial. J Clin Oncol. 2020;4:947–957. 10.1200/CCI.20.00081 PMC7768331 33112661

[38] Rodin G , Lo C, Rydall A, et al Managing Cancer and Living Meaningfully (CALM): a randomized controlled trial of a psychological intervention for patients with advanced cancer. J Clin Oncol. 2018;36(23):2422–2432. 29958037 10.1200/JCO.2017.77.1097 PMC6085180

[39] King A , Acquaye-Mallory A, Vera E, et al Feasibility and preliminary efficacy of a virtual reality intervention targeting distress and anxiety in primary brain tumor patients at the time of clinical evaluation: study protocol for a phase 2 clinical trial. BMC Cancer. 2023;23(262). 10.1186/s12885-023-10671-2 PMC10030076 36944930

[40] King A , Roche K, Leeper H, et al Feasibility of a virtual reality intervention targeting distress and anxiety symptoms in patients with primary brain tumors: interim analysis of a phase 2 clinical trial. J Neurooncol. 2023;162(1):137–145. 36884201 10.1007/s11060-023-04271-0 PMC9993385

