# Supplementary material for: The COVID-19 pandemic experience for patients with central nervous system tumors: Differences in patient-reported outcomes and practice recommendations
Source: Neurooncol Pract. 2024 Jul 19;12(1):76–86. doi: 10.1093/nop/npae067 (PMC11798606; doi:10.1093/nop/npae067)
Supplement: npae067_suppl_Supplementary_Materials [file npae067_suppl_supplementary_materials.docx]

**Supplementary Materials**

**Supplemental Table 1.** Reported symptoms on MDASI-BT and MDASI-SP at the moderate-severe level during and pre-COVID

|  | | COVID Year | Pre-COVID |
| --- | --- | --- | --- |
| MDASI-BT N | | 134 | 134 |
|  |  | N (%) | N (%) |
| Fatigue | | 36 (27) | 41 (31) |
| Feeling drowsy | | 27 (20) | 23 (17) |
| Difficulty remembering | | 35 (26) | 28 (21) |
| Disturbed sleep | | 20 (15) | 25 (19) |
| Feeling distressed | | 25 (19) | 25 (19) |
| Irritability | | 25 (19) | 19 (14) |
| Weakness on one side of body | | 23 (17) | 16 (12) |
| Feeling sad | | 18 (13) | 20 (15) |
| Difficulty concentrating | | 25 (19) | 19 (14) |
| Difficulty speaking* | | 24 (18) | 13 (10) |
| Pain | | 22 (16) | 14 (10) |
| Vision | | 21 (16) | 19 (14) |
| Numbness/Tingling | | 21 (16) | 16 (12) |
| Difficulty understanding | | 17 (13) | 13 (10) |
| Change in bowel pattern | | 18 (13) | 10 (8) |
| Dry mouth | | 21 (16) | 15 (11) |
| Lack of appetite | | 10 (8) | 14 (10) |
| Change in appearance | | 9 (7) | 7 (5) |
| Nausea | | 9 (7) | 13 (10) |
| Shortness of breath | | 8 (6) | 9 (7) |
| Seizures | | 6 (5) | 5 (4) |
| Vomiting | | 4 (3) | 5 (4) |
| Rash | | 4 (3) | 4 (4) |
|  | | COVID year | Pre-COVID |
| MDASI-SP N | | 23 | 23 |
|  |  | N (%) | N (%) |
| Fatigue | | 10 (44) | 9 (39) |
| Numbness/Tingling | | 8 (35) | 8 (35) |
| Pain* | | 8 (35) | 4 (17) |
| Weakness in arms/legs/trunk | | 6 (26) | 6 (26) |
| Disturbed sleep | | 2 (9) | 3 (13) |
| Sexual function | | 3 (13) | 5 (22) |
| Feeling distressed | | 2 (9) | 2 (9) |
| Radiating spine pain* | | 9 (39) | 3 (13) |
| Feeling drowsy | | 5 (22) | 5 (22) |
| Change in bowel pattern | | 5 (22) | 4 (17) |
| Loss of control of bladder/bowel | | 4 (17) | 4 (17) |
| Difficulty remembering | | 5 (22) | 4 (17) |
| Feeling sad | | 4 (17) | 1 (4) |
| Dry mouth | | 4 (17) | 2 (9) |
| Lack of appetite | | 0 (0) | 1 (4) |
| Shortness of breath | | 0 (0) | 1 (4) |
| Nausea | | 2 (9) | 2 (9) |
| Vomiting | | 1 (4) | 0 (0) |
| Rash | | 2 (9) | 1 (5) |

*Abbreviations*: NOB: Neuro-Oncology Branch; MDASI-BT: MD Anderson Symptom Inventory-Brain Tumor; MDASI-SP: MD Anderson Symptom Inventory-Spine Tumor

**p* < .05

**Supplemental Table 2.** Mood (PROMIS Anxiety and Depression) and cognition (NeuroQoL) assessments during and pre-COVID

|  |  | COVID Year | Pre-COVID |
| --- | --- | --- | --- |
|  | N | 149 | 149 |
|  |  | Mean (SD) | Mean (SD) |
| PROMIS Anxiety | T-score | 49.8 (9.9) | 49.1 (9.1) |
|  | None-mild  Moderate-severe | 87%  13% | 89%  11% |
| PROMIS Depression | T-score  None-mild  Moderate-severe* | 49.9 (9.0)  87%  13% | 48.1 (8.8)  92%  8% |
| PROMIS Anxiety & Depression | None-mild  Moderate-severe | 93%  7% | 96%  4% |
| NeuroQoL | T-score | 48.4 (11.2) | 49.5 (10.3) |
|  | Moderate-severe* | 24% | 18% |

*Abbreviations*: NOB: Neuro-Oncology Branch; SD: standard deviation; PROMIS: Patient-Reported Outcomes Measurement Information System

**p* < .05

**Supplemental Table 3.** General health status assessment on EQ-5D-3L during and pre-COVID

|  |  | COVID Year | Pre-COVID |
| --- | --- | --- | --- |
|  | N | 148 | 149 |
| Mobility | No problems with mobility | 68% | 65% |
|  | Some/Extreme problems with mobility | 32% | 35% |
| Self-care | No problems with self-care | 87% | 84% |
|  | Some/Extreme problems with self-care | 13% | 16% |
| Usual activities | No problems with usual activities | 60% | 52% |
|  | Some/Extreme problems with usual activities | 41% | 48% |
| Pain/Discomfort | No pain or discomfort | 62% | 59% |
|  | Moderate/Extreme pain or discomfort | 38% | 41% |
| Anxiety/Depression | Not anxious or depressed | 55% | 51% |
|  | Moderately/Extremely anxious or depressed | 49% | 45% |
| Health state | 11111 | 31% | 28% |
|  | non 11111 | 69% | 73% |
|  |  | Mean (SD) | Mean (SD) |
|  | Index score | 0.83 (0.16) | 0.82 (0.17) |

*Abbreviations*: NOB: Neuro-Oncology Branch; SD: standard deviation

**Supplemental Table 4.** Medication use reported at time of clinical evaluation

|  | COVID Year | Pre-COVID |
| --- | --- | --- |
| N | 138 | 141 |
| Medication Category | N (%) | N (%) |
| Anticonvulsants | 79 (58) | 81 (57) |
| Corticosteroids | 28 (20) | 31 (22) |
| Psychotropics | 47 (34) | 49 (35) |
| Supplements | 66 (48) | 66 (48) |
| Not assessed | 11 (8) | 8 (6) |

**Supplemental Table 5.** Stepwise logistic regression full and reduced models identifying significant predictors for moderate-severe depression for patients seen during COVID year (N=102)

|  | **Full Model** | | | **Reduced Model** | | |
| --- | --- | --- | --- | --- | --- | --- |
| Variable | OR | 95% CI | Sig | OR | 95% CI | Sig |
| Age at visit | 0.98 | .90, 1.06 | .580 |  |  |  |
| Sex  Female  Male | Reference  4.48 | –  0.62, 32.55 | –  .138 |  |  |  |
| Race  White  Other | Reference  0 | –  0, . | –  .999 |  |  |  |
| Ethnicity  Not Hispanic/Latino  Hispanic/Latino | Reference  3.69 | –  0, 2898.40 | –  .701 |  |  |  |
| Current tumor grade  Low grade (1 or 2)  High grade (3 or 4) | 21.49  Reference | 2.13, 216.50  – | **.009**  – | 14.12  Reference | 2.15, 92.59  – | **.006**  – |
| Active treatment at time of visit  No  Yes | Reference  2.11 | –  0.17, 26.00 | –  .561 |  |  |  |
| KPS  Good (≥ 90)  Poor (≤ 80) | Reference  1.42 | –  0.12, 16.62 | –  .781 |  |  |  |
| Progression status at visit  No progression  Progression | Reference  1.13 | –  0.08, 16.03 | –  .926 |  |  |  |
| Prior recurrence  No  Yes | Reference  0.30 | –  0.02, 4.12 | –  .366 |  |  |  |
| Fatigue severity  None-mild  Moderate-severe | Reference  3.22 | –  0.31, 33.80 | –  .330 |  |  |  |
| Distress severity  None-mild  Moderate-severe | Reference  32.17 | –  3.22, 321.08 | –  **.003** | Reference  25.72 | –  4.24, 155.87 | –  **< .001** |
| Disturbed sleep severity  None-mild  Moderate-severe | Reference  1.34 | –  0.14, 12.71 | –  .797 |  |  |  |
| Psychotropic medication use  No  Yes | Reference  7.33 | –  1.02, 52.46 | –  **.047** | Reference  5.52 | –  1.14, 26.79 | –  **.034** |

*Abbreviations*: OR: odds ratio; CI: confidence interval; Sig: significance level; KPS: Karnofsky Performance Status score

**Supplemental Table 6.** Stepwise logistic regression full and reduced models identifying significant predictors for moderate-severe depression for patients seen pre-COVID (N=128)

|  | **Full Model** | | | **Reduced Model** | | |
| --- | --- | --- | --- | --- | --- | --- |
| Variable | OR | 95% CI | Sig | OR | 95% CI | Sig |
| Age at visit | 0.91 | 0.82, 1.02 | .107 |  |  |  |
| Sex  Female  Male | Reference  2.47 | –  0.30, 20.51 | –  .402 |  |  |  |
| Race  White  Other | Reference  0.59 | –  0.01, 45.84 | –  .813 |  |  |  |
| Ethnicity  Not Hispanic/Latino  Hispanic/Latino | Reference  1.68 | –  0.00, 2305.81 | –  .888 |  |  |  |
| Current tumor grade  Low grade (1 or 2)  High grade (3 or 4) | 0.26  Reference | 0.03, 2.05  – | .200  – |  |  |  |
| Active treatment at time of visit  No  Yes | Reference  0.01 | –  0.00, 0.70 | –  **.035** |  |  |  |
| KPS  Good (≥ 90)  Poor (≤ 80) | Reference  10.64 | –  0.73, 155.09 | –  **.084** |  |  |  |
| Progression status at visit  No progression  Progression | Reference  1.87 | –  0.02, 218.31 | –  .797 |  |  |  |
| Prior recurrence  No  Yes | Reference  3.52 | –  0.20, 61.85 | –  .390 |  |  |  |
| Fatigue severity  None-mild  Moderate-severe | Reference  0.59 | –  0.04, 9.42 | –  .711 |  |  |  |
| Distress severity  None-mild  Moderate-severe | Reference  47.12 | –  1.88, 1183.43 | –  **.019** | Reference  30.46 | –  5.18, 179.08 | –  **< .001** |
| Disturbed sleep severity  None-mild  Moderate-severe | Reference  18.73 | –  0.77, 456.16 | –  **.072** |  |  |  |
| Psychotropic medication use  No  Yes | Reference  44.97 | –  2.14, 945.57 | –  **.014** | Reference  9.87 | –  1.59, 61.40 | –  **.014** |

*Abbreviations*: OR: odds ratio; CI: confidence interval; Sig: significance level; KPS: Karnofsky Performance Status score

**Supplemental Table 7.** Stepwise logistic regression full and reduced models identifying significant predictors for moderate-severe anxiety for patients seen during COVID year (N=102)

|  | **Full Model** | | | **Reduced Model** | | |
| --- | --- | --- | --- | --- | --- | --- |
| Variable | OR | 95% CI | Sig | OR | 95% CI | Sig |
| Age at visit | 0.94 | 0.87, 1.01 | **.087** |  |  |  |
| Sex  Female  Male | Reference  1.05 | –  0.20, 5.50 | –  .953 |  |  |  |
| Race  White  Other | Reference  0 | –  0, . | –  .999 |  |  |  |
| Ethnicity  Not Hispanic/Latino  Hispanic/Latino | Reference  1.80 | –  0.07, 46.57 | –  .722 |  |  |  |
| Current tumor grade  Low grade (1 or 2)  High grade (3 or 4) | 4.64  Reference | 0.70, 30.88  – | **.093**  – | 5.57  Reference | 1.12, 27.61  – | **.035**  – |
| Active treatment at time of visit  No  Yes | Reference  7.72 | –  0.83, 72.03 | –  **.073** | Reference  5.82 | –  1.05, 32.33 | –  **.044** |
| KPS  Good (≥ 90)  Poor (≤ 80) | Reference  0.29 | –  0.03, 3.10 | –  .309 |  |  |  |
| Progression status at visit  No progression  Progression | Reference  1.36 | –  0.15, 12.49 | –  .786 |  |  |  |
| Prior recurrence  No  Yes | Reference  0.69 | –  0.09, 5.23 | –  .718 |  |  |  |
| Fatigue severity  None-mild  Moderate-severe | Reference  3.23 | –  0.39, 27.06 | –  .279 |  |  |  |
| Distress severity  None-mild  Moderate-severe | Reference  13.63 | –  2.05, 90.65 | –  **.007** | Reference  10.57 | –  2.27, 49.17 | –  **.003** |
| Disturbed sleep severity  None-mild  Moderate-severe | Reference  0.10 | –  0.01, 1.95 | –  .129 |  |  |  |
| Psychotropic medication use  No  Yes | Reference  1.30 | –  0.27, 6.34 | –  .748 |  |  |  |

*Abbreviations*: OR: odds ratio; CI: confidence interval; Sig: significance level; KPS: Karnofsky Performance Status score

**Supplemental Table 8.** Stepwise logistic regression full and reduced models identifying significant predictors for moderate-severe anxiety for patients seen pre-COVID year (N=128)

|  | **Full Model** | | | **Reduced Model** | | |
| --- | --- | --- | --- | --- | --- | --- |
| Variable | OR | 95% CI | Sig | OR | 95% CI | Sig |
| Age at visit | 0.98 | 0.93, 0.324 |  |  |  |  |
| Sex  Female  Male | Reference  1.62 | –  0.45, 5.85 | –  .465 |  |  |  |
| Race  White  Other | Reference  2.64 | –  0.35, 19.79 | –  .344 |  |  |  |
| Ethnicity  Not Hispanic/Latino  Hispanic/Latino | Reference  1.36 | –  0.10, 18.65 | –  .819 |  |  |  |
| Current tumor grade  Low grade (1 or 2)  High grade (3 or 4) | 0.72  Reference | 0.19, 2.78  – | .637  – |  |  |  |
| Active treatment at time of visit  No  Yes | Reference  0.48 | –  0.09, 2.59 | –  .395 |  |  |  |
| KPS  Good (≥ 90)  Poor (≤ 80) | Reference  1.07 | –  0.20, 5.68 | –  .934 |  |  |  |
| Progression status at visit  No progression  Progression | Reference  1.25 | –  0.09, 17.57 | –  .869 |  |  |  |
| Prior recurrence  No  Yes | Reference  1.13 | –  0.26, 4.92 | –  .866 |  |  |  |
| Fatigue severity  None-mild  Moderate-severe | Reference  2.00 | –  0.41, 9.70 | –  .388 |  |  |  |
| Distress severity  None-mild  Moderate-severe | Reference  14.47 | –  2.34, 89.36 | –  **.004** | Reference  9.79 | –  3.12, 30.75 | –  **< .001** |
| Disturbed sleep severity  None-mild  Moderate-severe | Reference  0.40 | –  0.06, 2.94 | –  .371 |  |  |  |
| Psychotropic medication use  No  Yes | Reference  1.14 | –  0.30, 4.35 | –  .844 |  |  |  |

*Abbreviations*: OR: odds ratio; CI: confidence interval; Sig: significance level; KPS: Karnofsky Performance Status score
